# The phoenix of phonaesthetics: the rise of an old-new research paradigm on the beauty of language sound

**DOI:** 10.3389/fpsyg.2025.1720029

**Published:** 2025-12-05

**Authors:** Lukas Nemestothy, Vita V. Kogan, Susanne Maria Reiterer

**Affiliations:** 1 Department of Linguistics, University of Vienna, Vienna, Austria; 2 Vienna Doctoral School Cognition Behaviour and Neuroscience, Vienna, Austria; 3 Laboratory of Phonetics and Phonology, University of Lisbon, Lisbon, Portugal; 4 Vienna Cognitive Science Hub, Vienna, Austria

**Keywords:** speech perception, empirical aesthetics, sound symbolism, language attitudes and ideologies, aesthetic judgement, psycholinguistics, neuroaesthetics, music and language

## Abstract

This review traces the historical, cultural, psychological, and neuroscientific dimensions of phonaesthetics—the study of beauty in language sound. Once considered too subjective or ideologically charged for serious inquiry, the aesthetics of language is now re-emerging as a vibrant interdisciplinary field that draws on linguistics, psychology, cognitive science, neuroscience, aesthetics, and literary studies. This review offers a structured synthesis of current debates, theories, and empirical findings, while also outlining methodological innovations, including bibliometric mapping. Contemporary research demonstrates that sound is not merely a transparent medium for meaning but an aesthetic phenomenon in its own right, shaping how language is learned, remembered, and valued. Advances in cognitive science, neuroaesthetics and psycholinguistics have given new empirical grounding to questions once considered marginal. From David Crystal’s early discussions to modern work on sound symbolism, memory, and brand naming, evidence consistently points to the interplay between inherent linguistic values and culturally imposed norms. While aesthetic preferences differ across speakers and contexts, recurring patterns—such as the appeal of sonorous or rhythmic structures—suggest shared cognitive and emotional mechanisms. At the same time, language ideologies and the familiarity effect continue to modulate responses, underscoring the multifaceted reasons for aesthetic judgment. Reviving phonaesthetics therefore means more than cataloguing pleasant sounds. It invites renewed inquiry into why sound matters, how it contributes to identity, shapes evaluations of linguistic varieties, and allows speech to be experienced as art. In this light, language deserves recognition alongside music, painting, and literature as a legitimate subject of aesthetic appreciation. This review aims to highlight that speech sound can elicit emotion as powerfully as melody or color. The “phoenix” of phonaesthetics thus rises again—not merely as a study of linguistic beauty, but as a call to reimagine language as one of the arts.

## 1 Introduction

Phonaesthetics—the study of beauty in the sound of language—has often been dismissed as too subjective or culturally biased to be worth doing research on. As one popular aphorism in linguistics goes, “Before God and the linguists, all languages are equal.” Language is typically treated as a propositional system, primarily valued for its capacity to convey meaning and accomplish practical goals. Yet recent theoretical work comparing language and music introduced the propositional-aesthetic dimension, proposing that language can also be understood and studied as an artistic medium whose sound structures evoke affective and aesthetic responses (Haiduk and Fitch, 2022). In this framework, language typically occupies the propositional end of the propositional-aesthetic dimension, yet even ordinary speech can convey aesthetic value, revealing beauty as an intrinsic potential of linguistic expression. At the same time, advances in cognitive science, neuroaesthetics and psycholinguistics provide empirical tools capable of probing why and how speech sound elicits affective responses. These scientific advancements make it timely to revisit phonaesthetics not as an antiquarian pastime but as a contemporary, interdisciplinary field. Which posits phonaesthetics as the metaphorical “phoenix” which arises from obscurity to become a modern critical field of research.

Public discourse underscores the relevance of focusing on aesthetic perceptions of language. Internet forums frequently reveal strong lay opinions about the beauty of different languages. In one forum on reddit, a user remarks: “I like how my native language, Italian, sounds, but I’m highly biased of course […] I love how Greek sounds, that’s almost music” (r/conlangs, 2023 [2025]). Another user notes, “French and Russian for beauty […] Japanese is pleasant to listen to, but I would not use beautiful to describe it. Soothing maybe?” These examples illustrate that even popular opinion reflects a tacit awareness of both subjective taste and phonaesthetic dimensions. Within these snippets of the online discussion the users already perceive biases (L1), infer from the perception of language to neighboring domains in the arts (it sounds almost like music) and debate whether aesthetics in languages can be judged unidimensional (soothing instead of beautiful). These judgments already open the field for larger scale research into the beauty of language sound (Crystal, 2010; Reiterer et al., 2020).

This review therefore aims to combine the historical, cultural, psychological and neuroscientific strands that have contributed to the revival of phonaesthetics. Rather than argue for a single explanatory model, it aims to map the current landscape. A better understanding of phonaesthetics can inform second language learning and teaching (by addressing affective motivators to learning a new language), intercultural communication (by revealing biases that influence social outcomes), and applied domains such as brand design (where aesthetics shape user preference). Additionally, situating aesthetic judgement within cognitive and social frameworks helps explain why certain patterns recur across cultures, which might help shed light on the origin and purpose of human language. It also connects to broader questions of language evolution (Anikin et al., 2023) and to mechanisms of both first and second language acquisition (Reiterer et al., 2020). Most importantly, it contributes to the general understanding of aesthetic perception—how humans experience and evaluate beauty across different sensory modalities. However, it first and foremost aims to facilitate future research on phonaesthetics by providing an extensive overview of the status quo of research in this and neighboring fields.

Methodologically, the review follows an integrative approach, drawing together findings from linguistics, psychology, and neuroscience to outline the interdisciplinarity of phonaesthetics. Additionally, a targeted bibliometric mapping traces the hotspots of phonaesthetic research and aims to uncover linkages between the neighboring fields surrounding phonaesthetics. The review includes conceptual and historical approaches to phonaesthetics, empirical findings from psycholinguistic research, and neuroscientific perspectives. Each section highlights a different sub-field within the mosaic of phonaesthetic research.

In short, this review advances the claim that language should be considered as a domain of aesthetic experience within the arts combined. By channeling together diverse approaches and exposing productive strands—between inherent value and imposed norms (Giles and Niedzielski, 1998)—it aims to reframe phonaesthetics as a rigorous interdisciplinary field rather than a retreat into subjective opinions.

## 2 From Cratylus to Kiki-Bouba—sound symbolism revisited

This section seeks to delineate the field of phonaesthetics from “sound symbolism” and revisits foundational debates on the naturalness of linguistic signs, beginning with Plato’s language-philosophical dialogue “Cratylus”—where the question is posed to Socrates whether names are arbitrary or naturally suited to the things they describe (Fitch, 2016; Krapinger, 2014). This dialogue, featuring three philosophers, delves into the philosophical question of whether names possess an inherent motivation, link or association to the thing they name by nature (naturalness, not random, symbolic) or are merely products of convention through iterative speech community agreements and language use (arbitrariness, convention, random links). In Plato’s dialogue the three discussing philosophers are: Socrates, Cratylus and Hermogenes. Cratylus, the “naturalist,” holds that names imitate nature—that they are inherently correct representations of what they denote, driven by symbolism or iconicity (“nomen est omen”). By contrast, Hermogenes, the “conventionalist,” argues that words mean what they do simply because people agreed on them—i.e., arbitrariness. With these two positions, spoken in very basic terms, “sound symbolism, naturalness” and “arbitrariness, conventionalism” were born. Socrates at first leaned toward the naturalist view, but he remained uncertain throughout the dialogue and ultimately rejected both positions, lamenting that names and their referents provide too little and too unreliable evidence. Language being too “fuzzy,” one should just investigate the objects that are denoted.

In modern linguistics, however, Saussure’s arbitrariness is largely accepted and axiomatically counts as the norm perspective. However, iconicity resurfaces in phenomena like onomatopoeia and the Kiki-Bouba effect, where participants consistently match rounded shapes to the word “bouba” and spiky shapes to “kiki” (Ćwiek et al., 2021; Köhler, 1929; Ramachandran and Hubbard, 2001). A recent cross-linguistic study on guessing meaning from word sounds of unfamiliar languages (D’Anselmo et al., 2019) confirmed sound symbolism is independent of the mother tongue of the listener. These findings challenge strict arbitrariness, and as Fitch (2018) argues, show that the auditory system is tuned to certain regularities that evoke consistent affective responses. Within the last one and a half decades, sound symbolism gained ground again and resurged strongly within linguistic research (Sidhu, 2025) and paved the way for phonaesthetics research as well. However, it is surely still not as strong a position as arbitrariness. Metaphorically speaking, this could be seen as a kind of Hegelian dialectic tension field between “Socrates and Saussure,” meaning sound symbolism/iconicity versus arbitrariness, awaiting an intense era of future research in that field.

## 3 Mapping the landscape—a bibliometric analysis of phonaesthetics research

**Figure 1:**
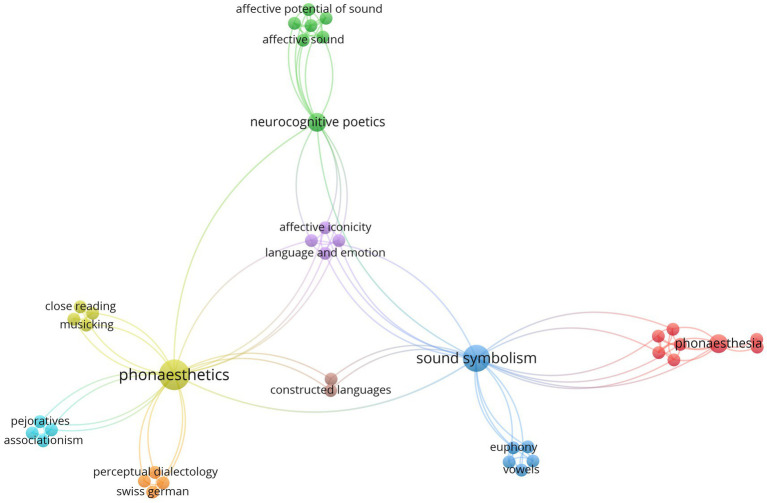
This visualized bibliometric network (VOSviewer; Van Eck and Waltman, 2014) shows the connections of the keywords chosen by the authors. The size of the circles indicates the number of mentions. Three key concepts can be highlighted: phonaesthetics, sound symbolism, and neurocognitive poetics.

This section reports a VOSviewer-based bibliometric analysis (Van Eck and Waltman, 2010) of publications on phonaesthetics. Using keyword searches in Scopus, we identify research clusters, publication trends, and conceptual networks to clarify how the field is evolving and to pinpoint gaps where interdisciplinary work is needed. To capture this niche domain, we queried nine search terms that had to appear in the title, abstract, or keywords—covering spelling variants of phon(o)(a)esthetic(s) and the adverb phonaesthetically (Crystal, 1995). The search yielded a corpus of 31 publications, underscoring both the field’s compact size and the opportunity for further research (Figure 1).

The first bibliometric network shows the analysis of the author’s chosen keywords. The three bigger circles show the keywords: phonaesthetics, neurocognitive poetics and sound symbolism. This network illustrates the interplay of phonaesthetics and its neighboring research fields of sound symbolism and neurocognitive poetics. On the one hand, sound symbolism examines perceptual correspondences between concepts and the speech sounds that denote them—how, and to what extent, a word’s sound can mimic or signal its meaning. More broadly, it explores mappings to perceived size, taste, smell, and other abstract domains, sometimes extending into emotional judgments—where its overlap with phonaesthetics is most evident. On the other hand, neurocognitive poetics focuses on how the brain processes the thoughts, language, music and imagery that arises from literature perception (Jacobs, 2015). The field of neurocognitive poetics clearly stresses the affective-aesthetic dimension which ties it to the field of phonaesthetics. The focus, however, is set on a strictly aesthetic use of language, i.e., poetry or, broadly put, literature, whereas phonaesthetics leaves it open to human vocal production in general.

A closer look at the keywords reveals the importance of affectivity in the field, alongside the perception of different languages and the related concept of phonaesthesia. It also shows the broadness of phonaesthetic research which is not only limited to standard and natural languages but encompasses research into language varieties and constructed languages as well.

**Figure 2:**
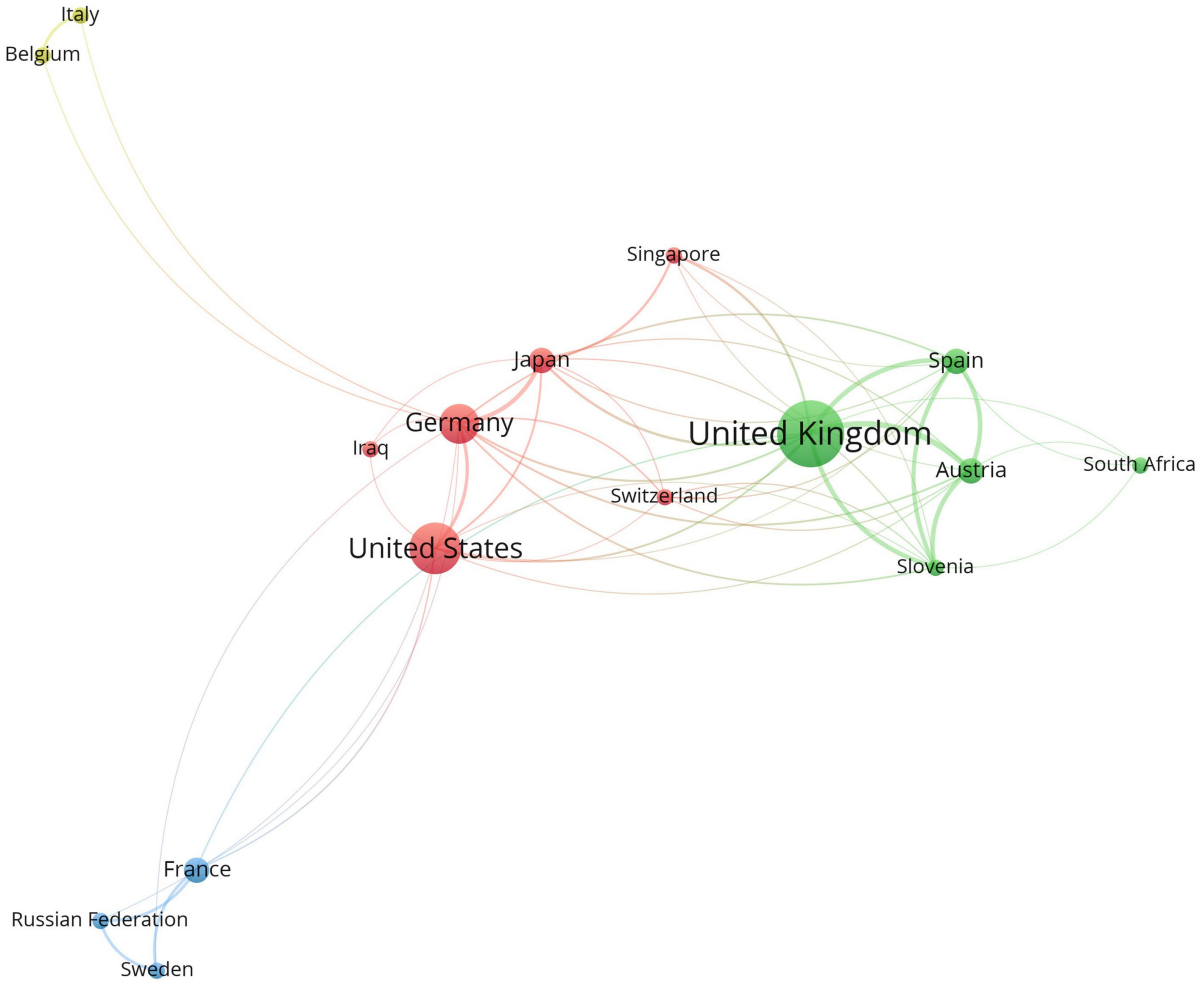
The network (VOSviewer; Van Eck and Waltman, 2010, 2014) shows the bibliographic coupling with countries as the unit of analysis. Seventeen countries had at least one document that fitted the search terms—one country, Jordan, has no connections and therefore has not been displayed in the network. The size of the circles displays the citations.

The bibliographic analysis on the countries of the universities/research institutions that the authors are associated with, has shown the rather typical WEIRD picture of modern research (Figure 2). The so-called WEIRD (“Western Educated Industrialized Rich Democratic”) bias encompasses multiple biases of both the samples investigated and the countries associated with researchers (Henrich et al., 2010). The network of the countries shows a strong tendency to western research—with big hubs in the United Kingdom, the United States, and Germany. Interestingly, Japan, Singapore, and South Africa are also bigger hubs in the network and might steer towards a more global understanding of research on phonaesthetics. This might challenge the Western part of the WEIRD bias in research, however it must be mentioned that these nations are also relatively rich, industrialized, democratic and educated.

In order to shine light on the “big neighbour” of phonaesthetics, a third bibliographic analysis was performed on sound symbolism. To capture this domain, we queried four search terms that had to appear in the title, abstract, or keywords—covering spelling variants of sound symbolism (soundsymbolism and sound-symbolism) and phono symbolism. The search yielded a corpus of 1,044 publications, setting it in stark contrast to the rather limited scope of phonaesthetic research of 31 publications.

**Figure 3:**
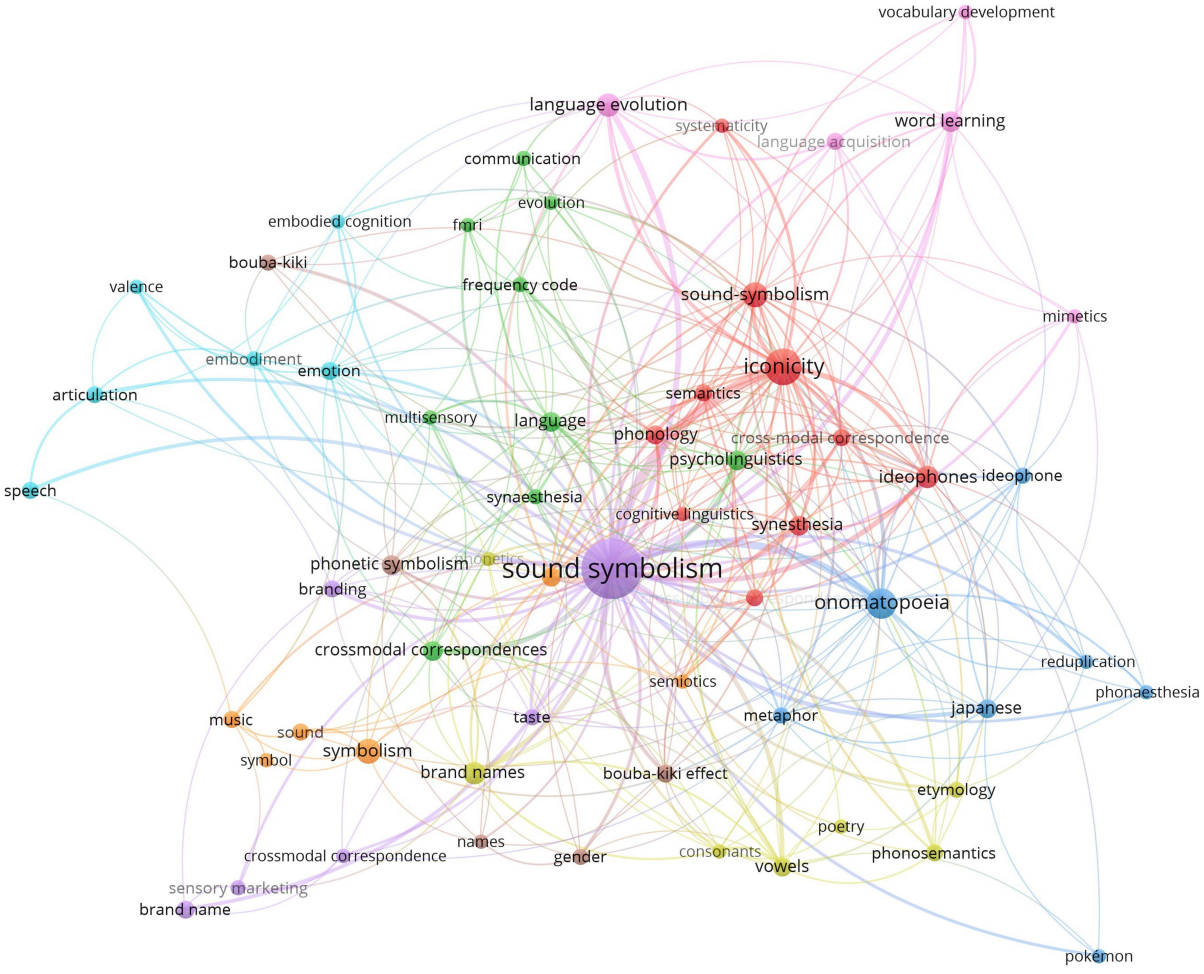
This visualized bibliometric network (VOSviewer; Van Eck and Waltman, 2014) shows the connections of the keywords chosen by the authors (with a minimum number of six occurrences). The size of the circles indicates the number of mentions. Three key concepts can be highlighted: sound symbolism, onomatopoeia, and iconicity.

The bibliometric network for sound symbolism (Figure 3) reveals a rich landscape of interrelated concepts. Several clusters emerge around theoretical, cognitive, and applied perspectives. Core linguistic fields such as phonology, semantics, and psycholinguistics are closely intertwined with cognitive and perceptual approaches that include synesthesia, embodiment, emotion, and multisensory processing. At the same time, applied and interdisciplinary domains such as brand names, sensory marketing and poetry demonstrate how sound symbolism extends beyond traditional linguistic research into areas of creativity and design. Its links to language evolution, language acquisition, and word learning highlights its relevance for fundamental questions about how meaning emerges from sound. The most prominent paradigm within sound symbolism research, the bouba-kiki effect, also appears as a well-integrated node. Additionally, onomatopoeia, which serve as typical examples for the connection between sound-image and concept, appear as a highly mentioned author keyword. Furthermore, the network shows clear overlaps with phonaesthesia and iconicity, echoing the connections observed in the bibliometric network on phonaesthetics (Figure 1).

In summary, these bibliometric analyses aim to give a comprehensive overview of the current landscape of phonaesthetic research, highlighting its interdisciplinary connections, geographic distribution, and conceptual breadth. The interplay between phonaesthetics, sound symbolism, and neurocognitive poetics underscores the field’s potential to bridge linguistic, cognitive, and aesthetic domains. Considering that there are a thousand more publications addressing sound symbolism than phonaesthetics under comparable search criteria, this comparison reinforces the view that phonaesthetics represents an emergent and still-developing field, whereas sound symbolism constitutes a more established and extensively connected research domain. However, the analysis also reveals significant gaps, particularly in the diversity of research contexts and the rather limited non-WEIRD perspectives. By addressing these gaps, future research can deepen the understanding of the aesthetic and affective perceptions of language.

## 4 Between imposed norm and inherent value

**Figure 4:**
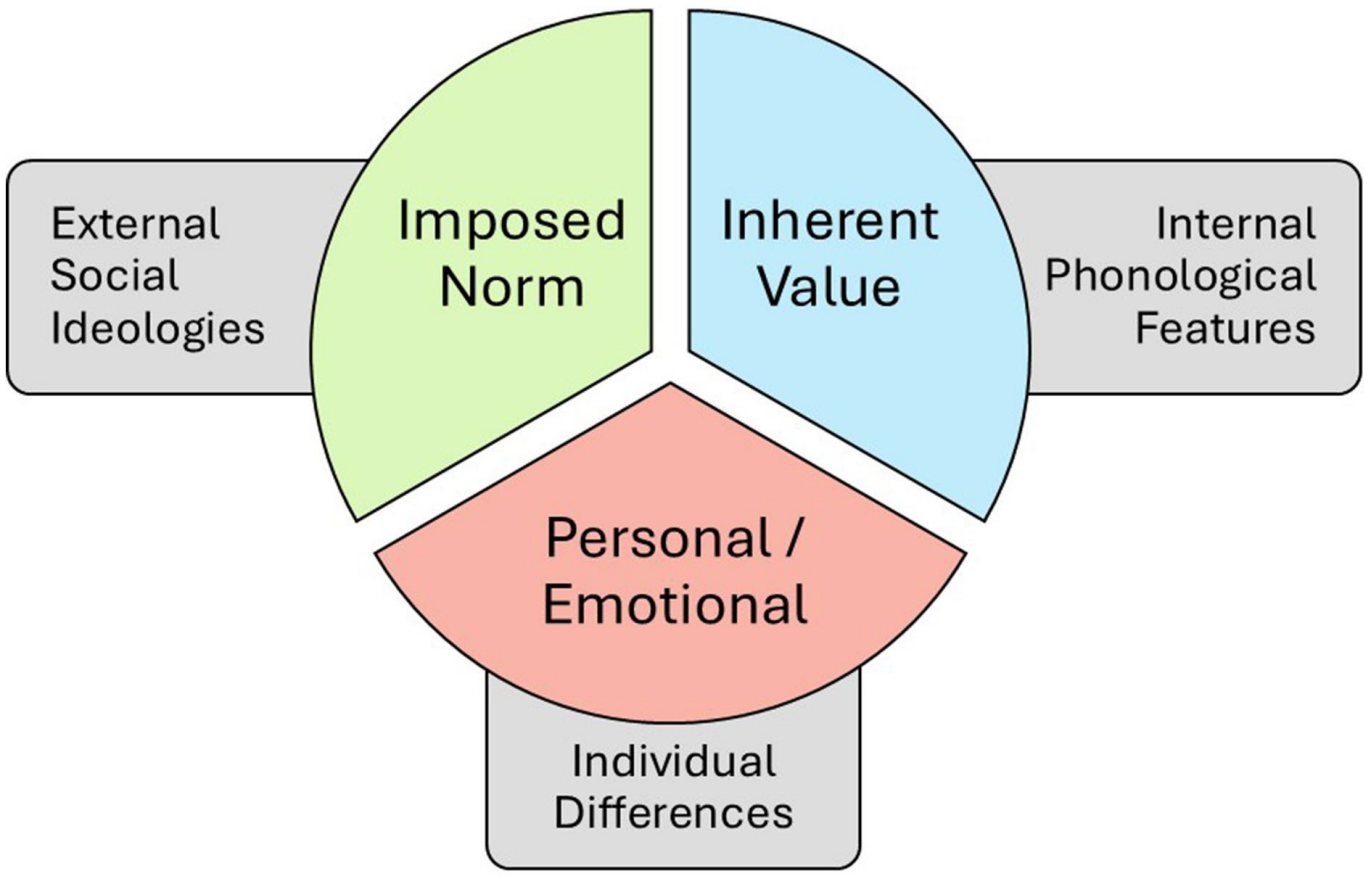
Influences on language perception. This figure illustrates the three main influences on the phonaesthetic ratings of language. Imposed norm highlights the influence of external social ideologies and how they alter the way a language is judged, since a language is inextricably intertwined with the associated culture. Inherent value summarizes all approaches of internal linguistic features and how these shape the perception. Personal and emotional influences are added to this model since on an individual scale (i.e., per rater) languages are also influenced by the personal biographies of listeners.

Perceptions of language beauty are shaped by both external social ideologies (imposed norm) and internal phonological features (inherent value). As Giles and Niedzielski (1998) point out, “What listeners hear is shaped not only by the acoustic signal but by who they think is speaking.” Cultural stereotypes, such as German being “harsh” or French being “romantic,” often override phonetic perception (Figure 4).

Imposed norm and inherent value are not mutually exclusive but rather interconnected, forming a multifaceted framework through which languages are judged. While the hypothesis of imposed norms highlights the role of cultural stereotypes and societal structures in shaping linguistic preferences, inherent value focuses on the intrinsic qualities of language (Giles and Niedzielski, 1998).

Beyond imposed norm and inherent value, personal and emotional factors play a crucial role in shaping linguistic aesthetics. An individual’s familiarity with a language can significantly influence their perception of it. As per the mere-exposure effect, people develop a preference for things only because they are familiar with them (Zajonc, 1968). This phenomenon can be explained through both evolutionary and cognitive frameworks: familiar stimuli are seen as safe and favorable, while repeated exposure facilitates processing, enhancing aesthetic pleasure. When empirically testing for familiarity, Reiterer et al. (2020) found that about 40%–50% of the overall variance in their phonaesthetic studies was explained by the factor of familiarity to a language. However, the relationship is not straightforward: While familiar languages are generally preferred, native languages (L1s) or those closely related to them did not receive the highest ratings. Instead, individuals favored foreign languages with an “exotic touch,” particularly those they have learned as second languages. Similarly, positive interactions with speakers of a particular language can alter one’s perception of the culture associated with a language and therefore also influence the ratings of it. Furthermore, a language may evoke pleasant memories or feelings of nostalgia, making it more appealing to the listener. Negative experiences can lead to unfavorable judgments. The personal and emotional influences underscore the subjective nature of linguistic aesthetics, revealing how deeply intertwined they are with individual biographies and social contexts. Interestingly, even personality plays an influencing role. Winkler et al. (2023) could show that the big-five personality dimension “emotional stability / neuroticism” played a significant role in how intensely people rated languages on the eroticity dimension. People with higher scores in emotional stability also rated languages more intensely on the eroticism scale.

Imposed norm refers to the societal and cultural frameworks that influence which languages are considered prestigious or desirable. These norms are often rooted in historical, political, and societal contexts, where the language of dominant groups is privileged through mechanisms of education, media, and legislation. For example, Standard British English and Parisian French have historically been elevated due to their association with centers of power, culture and commerce. Had these centers been located elsewhere, the linguistic varieties associated with them might have been trivialized instead of celebrated. Cultural stereotypes play a significant role in the imposition of linguistic norms. German, for instance, is often perceived as harsh and strict, a stereotype that aligns with broader cultural associations of Germany as a disciplined and industrious nation. In contrast, Italian is often referred to as romantic and elegant (Giles and Niedzielski, 1998).

While the imposed norm emphasizes the external influences on aesthetic ratings of languages, the concept of inherent value focuses on the internal, linguistic qualities of languages. Proponents of the inherent value hypothesis argue that certain sound patterns are universally pleasing, regardless of culture or history—for example, a high share of high vowels, greater sonority, and many open syllables (Crystal, 1995). Yet observed variability indicates that, even if some sounds possess intrinsic appeal, their reception is still shaped by individual differences and cultural context.

Irvine and Gal (2000) defined three semiotic processes which explain how linguistic features become ideologically loaded: Iconization describes the transformation of linguistic features of a social group that are used as an iconic representation of the group. It functions as if a linguistic feature depicts the inherent nature of the group. Fractal recursivity describes the process of projecting and reproducing an opposition, which is significant on one level of analysis, at other scales—which reinforces identities and social distinctions. Erasure describes the process in which an ideology renders some people or groups invisible. For example, a language might be described as being homogeneous, while the internal variations (e.g., regiolects/sociolects) are disregarded.

Shifting the focus from imposed norm to inherent value, research suggests that certain phonetic and prosodic characteristics reliably enhance the perceived pleasantness of a language. For instance, voiced consonants, smooth prosody, and syllabic regularity tend to be judged as more appealing (Reber et al., 2004). Beyond these factors, features such as faster speech tempo and reduced pitch variance have also been shown to contribute to positive evaluations (Kogan and Reiterer, 2021). Other studies point to the role of sonority, vowel share, timing properties, and the overall consonant-vowel (CV) structure as further determinants of attitudes towards language (Reiterer et al., 2020). More recent findings show that a higher share of back vowels influence the aesthetic responses of listeners negatively (Nemestothy et al., 2024). Taken together, these results highlight the multifaceted nature of inherent value in language perception, revealing how a complex interplay of segmental and suprasegmental features influences aesthetic judgments.

### 4.1 Language ideologies

Phonaesthetics also offers a concrete entry point into language ideology research, revealing how aesthetic judgments about sound are produced, circulated, and justified across interactional, discursive, and experiential levels. In language ideology research, three central perspectives are distinguished: the interactional perspective (linguistic anthropology), the discourse perspective (critical sociolinguistics), and the subjective/experiential perspective (language biography research) (Busch, 2019). These perspectives reveal how language ideologies operate at multiple levels—shaping interaction, discourse, and embodied subjectivity. Language ideologies can be defined as bundles of evaluations and beliefs about language and language use that justify communicative practices. These ideologies manifest both in explicit public debates (e.g., debating the use and frequency of loanwords) and in subtle everyday judgments during interaction. They are not mere “distortions” but integral parts of communication (Busch, 2019).

Silverstein (1979) conceptualizes linguistic ideologies as the metapragmatic beliefs speakers hold about language—beliefs that not only describe but actively shape communicative practice. He argues that speakers’ notions of what language *is* and how it *should* be used function as rationalizations of perceived linguistic structure and behavior. While such everyday “theories” of language may occasionally coincide with scientific linguistic analyses, they typically arise within distinct social and cultural systems of meaning. From this perspective, ideological evaluations do not merely accompany language use, they feed back into it. This influences how variants gain or lose prestige and, over time, drives processes of language change. This focus on the micro-interactional negotiation of linguistic value parallels approaches that explore how aesthetic and emotional responses emerge during speech perception. However, ideologies operate beyond the interpersonal level, as shown by Busch (2019) where language becomes bound to political projects of nationhood, standardization, and control.

In contrast to arguing about the complexity and the micro level of negotiations between hierarchies and assigned status to various linguistic varieties, it seems as if these hierarchies are rather stable. For the context of Britain, Sharma et al. (2022) investigated the change over 50 years towards the attitudes to various British accents and dialects. They concluded that the attitudes are remarkably stable. Hence, the status of accents seems to be long-standing and, in a sense, engraved in a society.

The theory of Bourdieu’s (1977) linguistic marketplace describes how certain ways of speaking acquire value within a given social space. Just as in an economic market, languages are evaluated depending on how closely they align with the worth the society ascribes to them. This means that speakers constantly negotiate their position by adapting their language to what is socially acceptable or prestigious. The “linguistic capital” determines how their status is perceived. In contrast, Foucault’s (2008) concept of *governmentality* emphasizes how power operates not only through laws and institutions but also through subtle forms of self-regulation. Language ideologies become effective precisely because individuals internalize norms of correctness and appropriateness, shaping their own behavior in line with the broader discourse. Taken together, these concepts highlight how imposed norms work simultaneously from the outside (regulation of markets) and from within (self-disciplining).

The points mentioned above mostly align with the concept of imposed norm. However, ideologies are not only external influences stemming from societal discourse but also embodied in the individual. Busch (2017) emphasizes the concept of “Spracherleben” (the lived experience of language). Through lived language experience the ideological positioning towards language gains an emotional dimension. Therefore, ideologies are not only rationalized judgments but also affective experiences. To underscore the importance of the lived experience of language we highlight some excerpts from participant feedback of Kogan and Reiterer’s (2021) study on phonaesthetic attitudes across languages. For example, this remark about Welsh: *“I spent a year in Wales and may have some strong (positive) emotions towards this language.”* In this example, the participant is reflecting their personal, individual “Erleben,” history and familiarity with the language. Another participant explained their lower rating of Welsh by attributing it to the language’s inherent sound qualities: “*Soo many back / uvular sounds.*” Another informant, by contrast, wrote: “I like [Spanish] probably because of positive associations with Spain and Spanish people (less so because of language-specific characteristics),” illustrating how internalized norms or stereotypes can shape evaluations. Feedback on Italian echoed the inherent-value view: “Open syllables make it seem very pleasant to me.” A comment on Greek reflected a pure stereotype: “Feels like summer.” And some remarks were explicitly personal and affective, such as for French: “Calming; I even experienced slight ASMR [‘chills’]”—or simply playful, as with Icelandic: “Funny language.” This mirrors the framework of Figure 4: people orient to societal norms (imposed norm) and to personal affective reactions (personal and emotional influence) and to inherent values.

### 4.2 Language attitudes

Language attitudes are understood as evaluative orientations toward languages and their speakers. These evaluations can be conscious or subconscious, positive or negative, and they shape social interactions. Language attitudes can be defined as positions (in the form of reactions, judgments, assessments, evaluations, associations) regarding language and speech and their speakers (Soukup, 2019). This highlights that attitudes are not only beliefs but embodied reactions with an emotional component (Busch, 2017), which can manifest affectively and can be measured. Forms of measurement include: Likert scales, semantic differentials, response latency measures (e.g., the Implicit Association Test). Additionally, there is also a tradition of physiological measures, such as skin conductance rate/galvanic skin response or facial muscle activity, used to capture affective components of attitudes and evaluations.

The matched-guise technique (Lambert et al., 1960) has been highly influential in language-attitudes research. In this method, the same speaker produces different language varieties (or “guises”), and listeners rate the recordings on traits such as intelligence, friendliness, and attractiveness. By holding the speaker constant, the design isolates language as the causal factor in evaluation. In the original Montreal study, both English- and French-speaking participants consistently rated the English guises more favorably across multiple scales (e.g., attractiveness, intelligence, kindness). Lambert et al. (1960) noted that it was unsurprising for English speakers to rate members of their own linguistic group more favorably; the novel finding was that French speakers did so as well. This pattern likely reflects the perceived higher status of English in Montreal. Strikingly, French participants rated French guises less favorably than English participants did, highlighting the interplay of societal hierarchies and self-regulation, aptly captured by Foucault’s notion of governmentality (i.e., internalized governance of conduct)—here, valuing a higher-status variety that is not one’s own. Consistent with this, Kogan and Reiterer (2021) found that participants did not prefer their native languages. They argue that familiarity associated with foreign/second-language learning—the so-called “exotic touch”—shapes aesthetic judgements: the more languages participants spoke, the more they reported enjoying the sound of foreign languages.

The matched-guise technique stems from a distrust in people’s overtness to rate or judge a language, either consciously or sub-consciously. The assumption is that in a questionnaire people would have a favorable picture and they would not share their “true” attitudes. The basis is the assumption that different languages/language varieties trigger certain social categorizations that will lead to a set of group-related trait-inferences. In other words, hearing a voice that is classified as *French-Canadian* will predispose listeners to infer that they have a particular set of personality-attributes. In the matched guise-technique special care is needed to ensure that the guises are perceived to be authentic, since the speakers should not be identified as being bi- or multilingual. Prosodic and paralinguistic features of voice, such as pitch, voice quality, and speech rate are kept constant as far as possible across the different recordings (Giles and Billings, 2004). If the matched-guise technique is applied correctly it elicits the reactions to the speakers based on linguistic cues (inherent value based) and the entailed categorizations (imposed norm based) without any interferences of the individual voice qualities.

Hilton et al. (2021) used the matched-guise technique to investigate the differences in judgements and attitudes to Swedish and Danish. In their case, the listeners were students from China with no prior exposure to either language. The findings indicate that these listeners evaluated Swedish as sounding more pleasant than Danish, which suggests that certain linguistic features may carry cross-cultural appeal (Schüppert et al., 2015). They point out that the prosodic features are an influence for the difference in the aesthetic judgements. This example highlights the possibilities of investigating inherent value based influences on speech perception.

A common variant of the matched-guise technique is the verbal-guise method, a simpler design in which different speakers provide the stimuli for different languages or varieties—eliminating the need for bilingual or multilingual speakers and making it especially practical for studying language varieties (Chan, 2021). Another variant is the open-guise technique, where participants are told that the different guises come from the same speaker; this removes the element of deception that drew criticism of the original method. Studies show that open-guise designs can also robustly reveal language attitudes (Soukup, 2021) and are useful for simulating real-life code-switching, allowing researchers to test the communicative effects of switching forms within a single interaction.

This dichotomy of trusting the “honest souls” of participants in reporting their attitudes versus the felt need to deceive participants when attitudes are elicited, builds the basis of the following two models. On the one hand the cognitive-psychological model defines attitudes as stable, latent predispositions. This model assumes internal structures composed of cognitive, affective, and behavioral components. On the other hand, the constructionist model defines attitudes as not pre-formed but emerging as *interactional practices* (performed). Attitudes are seen less as mental objects and more as contextually situated evaluations. This model is explained in Potter (1998) as follows:

“That is, it assumes that people have some inner position - the attitude - but that it varies over time. Discursive social psychologists have not started with the assumption that people must have consistent personally held evaluations that are carried from one context to another. The variability they are interested in is not a temporal fluctuation like changes in the weather, cloudy for a few days and then sun. Rather it is a discrete and specific variation tied to the nature of the action that is being performed. The point again is that evaluations are not treated as something that are carried around ready made by participants but are worked up in a way that is suitable for what is being done. In discursive social psychology attitudes are performed rather than preformed. The second problem is that it treats people as ‘honest souls’ who can and will provide comprehensive, honest and reliable information about their actions and mental states. […] The apparatus of traditional attitude measurement relies much more centrally on people being honest souls than its discursive alternative does.”

(Potter, 1998, p. 19). This focuses the debate on attitude towards language as a “trait versus state” debate and highlights the potential fluency in ratings and attitudes of language. Considering that in the constructionist model the attitudes are subject to change, this would influence the way attitudes would have to be researched. Soukup (2019) notes that language attitude research has always occupied a paradoxical position: While often adopting the cognitive definition from psychology, in practice it treats attitudes as multidimensional and socially embedded.

To conclude the subsections on language attitudes and language ideologies we can summarize their similarities. Research in both fields shares common ground in examining the social meanings attached to language, but they differ in their focus and methodological traditions. Both fields are concerned with how speakers evaluate, perceive, and orient themselves toward varieties of language, accents, or registers, and both acknowledge that such evaluations are not only influenced by linguistic features (inherent value) but entangled with issues of identity, power, and social belonging. However, while language attitude studies typically emerged from social psychology and applied linguistics, using experimental or survey methods to measure individuals’ evaluative responses toward languages or speakers, language ideology research is rooted in linguistic anthropology and sociolinguistics, emphasizing the historically and culturally embedded belief systems that rationalize and naturalize these evaluations. In other words, attitudes are often approached as individual-level, measurable dispositions, whereas ideologies are understood as collective, discursive frameworks that shape and constrain those dispositions.

Empirical work on phonaesthetics has sought to reduce culturally imposed norms by presenting listeners with lesser-known or unknown languages (Kogan and Reiterer, 2021; Reiterer et al., 2020; Winkler et al., 2023) or constructed languages (Mooshammer et al., 2023), aiming to approximate “inherent” aesthetic preferences. Early results show that this manipulation markedly weakens familiar stereotypes—most notably the “Latin Lover effect” (a Romance-language advantage on beauty/eroticity ratings; Reiterer et al., 2020)—because familiarity strongly boosts liking: when a language is recognized, it is usually rated more positively. The opposite direction does exist as well, but is much less pronounced (Nemestothy et al., 2024; Winkler et al., 2023; Gelitz, 2025). That said, it remains unclear whether the Latin Lover effect can be entirely explained by imposed norms. Familiarity, language ideologies, and stereotypes are related but distinct constructs that require further empirical disentangling in future phonaesthetic research. Furthermore, research needs to identify possible other driving forces/factors as well, which to date are still not discussed.

## 5 The aesthetics of constructed languages and pseudowords

Since J. R. R. Tolkien—Oxford philologist and author of *The Lord of the Rings*—is often credited with popularizing the term phonaesthetics, it is natural to examine constructed languages (conlangs) both as an object and an outcome of phonaesthetic design. Tolkien’s own Elvish languages (e.g., Quenya, Sindarin) were crafted to sound beautiful according to his aesthetic ideals. Similarly, modern conlangs such as Dothraki (from *Game of Thrones*) are purpose-built systems in which intentional design choices on the sound pattern of the language aim to convey a distinct picture to the audience.

Most notably, the study by Mooshammer et al. (2023) delves into the field of phonaesthetics in conlangs, exploring how the sound structures of these fictional languages influence listener perceptions. The research is grounded in two primary aims: first, to determine whether listeners rate conlangs in alignment with the impressions intended by their creators (e.g., pleasant vs. unpleasant, good vs. evil, peaceful vs. aggressive), and second, to investigate whether these ratings correlate with specific phonetic and phonological characteristics of the languages.

In the case of conlangs we have a feature which is not present in most of phonaesthetics research, however, crucial in the field of empirical aesthetics and creativity research: the creator. As Brown (2024) shows with the creativity-aesthetics cycle reframed from the standpoint of the social roles, there are two crucial roles: creators and consumers. The former imbue their creative products (in this case conlangs) with features that should make them aesthetically appealing to the consumers. The latter select the products that they find aesthetically pleasing and give responses or judgements on the aesthetics of certain products which then influences the creators again. For example in the Elvish languages, Quenya and Sindarin, sonorous vowels and specific syllable structures are used to create a sense of beauty and peace.

Aesthetically pleasing, in this context, does not necessarily mean universally beautiful—it may also refer to novelty or, in the case of constructed languages, to sound features intentionally designed to set a particular mood or atmosphere. Having the role of the creator clearly filled gives the research on conlangs an advantage, because the intentions of the creators are often well-documented and in some cases the creators are still able to be contacted. Mooshammer et al. (2023) also highlight examples where the creators deliberately use sound symbolism to evoke negative impressions. For example, harsh guttural sounds, voiceless fricatives, and stops are often employed to convey aggression or malevolence, as seen in Klingon and Orkish.

The main findings of Mooshammer et al. (2023) reveal that listeners indeed form consistent impressions of conlangs based on their sound structures, even when the stimuli are presented without emotional intonation or sound effects. For instance, Klingon and Dothraki were rated as more unpleasant, aggressive, and evil, aligning with their creators’ intentions, while Elvish languages like Sindarin and Quenya were perceived as pleasant and peaceful, as the creator, J. R. R. Tolkien had envisioned. Interestingly, Orkish and Khuzdul, which were expected to sound harsh and negative, were rated more positively than anticipated, suggesting potential methodological limitations or the influence of participants’ native language on their evaluations.

The study also identified specific phonological and phonetic features that influenced listener ratings. Languages with a higher percentage of voiced sounds and lower pitch were rated more positively, while those with a greater proportion of non-German sounds were perceived as more unpleasant, emphasizing the role of “otherness” in shaping perceptions. However, contrary to expectations, features like sonority and the presence of back vowels did not significantly predict impressions, challenging some prior assumptions about phonaesthetic universals.

The study supports the inherent value hypothesis, which posits that the intrinsic phonetic and phonological properties of a language influence its perception, independent of social or cultural associations. This is particularly evident in conlangs, where listeners lack prior familiarity or imposed norms, allowing the sound structure to take center stage in shaping impressions. While the results affirm the role of phonaesthetics in conlangs, the study acknowledges certain limitations. For example, the influence of participants’ native language on their evaluations cannot be entirely ruled out. Nonetheless, the findings provide valuable insights into how inherent values of sound contribute to the aesthetic and emotive impact of languages.

To probe the “constructed” side of language, researchers also use pseudowords. Lev-Ari and McKay (2022) examined sound symbolism in what they called swearing (“the sound of swearing”) and showed that some phonemes are better suited to conveying offensiveness and harshness than others. Across typologically distant languages, approximants are underrepresented in swear words—apparently symbolizing calmness/inoffensiveness. Experiments confirmed this: participants were less likely to label pseudowords with approximants as swear words (e.g., juxtaposing approximants to affricates, “sola” was judged less offensive than “sotsa”). Additionally, sanitized versions of English swear words (“minced oaths”) often introduce approximants, further supporting their perceived inoffensive quality. Examples for “minced oaths” show various ways of influencing the perceived inoffensiveness, for instance introducing more voiced consonants, e.g., in English, transforming “fucking” to “frigging” or “effing”; in German, transforming “Scheiße” to “Scheibe[nkleister].” In a related vein, Aryani et al. (2018) found that short vowels, voiceless consonants, and voiceless sibilants sound more arousing and negative (e.g., “piss” is perceived as ruder than “pee”). Together, these results point to a cross-linguistic cognitive bias in the phonaesthetic properties of sounds, extending sound symbolism from individual word meanings to broader pragmatic functions like emotional expression and social interaction.

This section’s findings provide empirical evidence supporting the phonaesthetic logic behind language construction for narrative effect. Essentially, creators of conlangs must carefully design and adjust phonetic sound patterns to evoke specific emotional responses from their audience. The study of constructed languages, particularly because they have identifiable creators, offers a unique and valuable perspective on the aesthetics of language. This research sheds light on how intentional phonetic design can shape emotional and narrative impact.

## 6 Phonaesthetically speaking: pleasing words from research to marketing

British linguist David Crystal popularized the term phonaesthetics and helped establish it as a field, greatly inspiring later researchers with his work. In his influential essay *“Phonaesthetically Speaking”* (Crystal, 1995), he framed phonaesthetics as the study of the aesthetic aspects of language sound—arguing that sound is not purely arbitrary—and highlighted its importance not only in literature but also in cultural identity and pronunciation. Across later work (e.g., Crystal, 1999, 2010), Crystal contends that sounds, words, and sentences can carry aesthetic and iconic value across genres; that languages exhibit distinct phonaesthetic profiles; and that everyday language use, not just literary style, involves matters of taste. He situated phonaesthetics at the intersection of phonetics, phonology, stylistics, and lexicography, showing how sound contributes to meaning and identity. Challenging strict versions of linguistic arbitrariness, he maintains that perceived sound beauty is shaped by language-specific phonetic/prosodic patterns and by cultural familiarity (a factor later supported empirically; see Reiterer et al., 2020).

In *“Phonaesthetically Speaking,”* Crystal also proposed a systematic matrix for analyzing phonaesthetic effects, including the frequency and types of consonants and vowels (manner/place of articulation), distribution patterns, clusters and collocations, syllable counts, and stress patterns. He illustrated the approach with words often cited as especially pleasing—e.g., *cellar door* (famously a “list leader”), *lullaby*, *velvet*, *whisper*, *melody*, *bobolink*—while noting that negatively valenced words can still sound appealing (e.g., *peril*, *tremulous*, *phlegmatic*, *flatulent*). From these observations, he distilled practical criteria that function as guidelines for evaluating sound aesthetics.

“…it is possible to see how we can create phonaesthetically pleasing new words. It would seem advisable to give them three syllables, to stress the first syllable, to use at least one /m/ or /l/ (preferably both), to introduce high-frequency consonants and avoid low-frequency ones, to have at least three different manners of consonantal articulation, to keep the vowels short, and to have the vowels move from mid towards high, and from front towards back.” (Crystal, 1995, p. 11).

In our view this recipe-like prescription of how to create beautiful sounding words must have found myriads of imitators in commercial and pharmaceutical products to date. Other than applying these criteria in real world applications, research until this day continued partly to use these early findings and build on the questions and material he described (Lev-Ari and McKay, 2022; Matzinger and Kosic, 2025a; Mooshammer et al., 2023). Contemporary research in phonaesthetics increasingly adds experimental, quantitative, acoustic, psycholinguistic and psychophysiological methods to the observations Crystal popularized (Kogan and Reiterer, 2021; Matzinger and Kosic, 2025a, 2025b; Mooshammer et al., 2023; Nemestothy et al., 2024; Winkler et al. 2023).

Drawing on Crystal’s phonaesthetic pleasantness hierarchy, Matzinger and Kosic (2025a) tested how phonetic composition—and thus phonaesthetic beauty—shapes a word’s appeal, memorability, and learnability. They manipulated pseudoword phonemes (e.g., “smanious” for the appealing condition, “creetious” for the intermediate condition, and “gruhious” for the unappealing condition) and collected both, appeal ratings and recall. The key result: phonaesthetic value boosted memory overall. Intriguingly, however, the intermediate items received the highest appeal ratings by the participants in this study, contrary to the designers’ intentions. The authors argue that these findings link aesthetics to cognition by empirically connecting phonaesthetic judgments with memory, with implications for language learning, marketing, and language evolution (where sound shape may influence the retention and diffusion of forms: e.g., Anikin et al., 2023).

The same research group (Matzinger et al., 2021) showed that aesthetically pleasing prosody facilitates speech segmentation. Listeners rated isochronous words and items with syllables lengthened or shortened at the beginning, middle, or end; prosodic patterns that were preferred aesthetically were also those that best supported segmentation. This suggests a link between phonaesthetic preference and language processing/learning, with implications for language acquisition and the diachronic stability of prosodic patterns.

The ability to “speak phonaesthetically” has a long-standing, practical history and is exploited in brand naming within marketing and consumer psychology. Successful brand names often craft appealing pseudowords that align with consumers’ phonaesthetic preferences. Practitioners have long leveraged the link between sonority and auditory pleasure: open vowels like /a/ and /o/ recur in iconic names—*Zara, Prada, Coca-Cola, Honda, Duron, Toto, Volvo, Sony, Kodak*. Rolex’s founder even pursued an onomatopoetic effect, intuitively choosing /r/ and /l/—among the most sonorous consonants—to suggest the watch’s smooth, rounded form (Feng, 2016).

Beyond intuition, research shows that phonetic makeup shapes perceived brand personality and value. Klink and Athaide (2012) report that back vowels tend to convey a rugged personality, whereas front vowels signal sincerity and sophistication. Pogacar et al. (2018) find that people implicitly and explicitly prefer sounds common in top brand names—e.g., names with /s/ (such as “simal”) over those with /θ/ (“thimal”)—and that these preferences correlate with a greater willingness to pay. Together, these findings suggest that the prevalence of certain sounds among leading brands reflects systematic attitudes toward phonetic features, making phonaesthetics a strategic tool for naming and positioning.

Studies consistently show that the sounds in brand names—especially vowel placement (front vs. back)—shape perceptions of product attributes (e.g., size, softness, richness) and influence preference, recall, and purchase intentions. Names whose sounds fit the product are liked and remembered more (Brennan et al., 2024; Klink, 2000; Lowrey and Shrum, 2007; Yorkston and Menon, 2004). These “semantically congruent” phonaesthetic names (e.g., *Snyre* for a nose spray, but not a car) boost processing fluency, yielding more favorable judgments and behaviors (Brennan et al., 2024). The effects are robust across languages (Shrum et al., 2012) and reinforced by memorability findings (Boltz et al., 2016). In short, phonaesthetics—and phonaesthemes—are actively leveraged to craft appealing, memorable, and competitive brand names.

## 7 Poetry and the poetic function of communication

One of the most compelling domains in which to explore the aesthetic appeal of language is poetry. Roman Jakobson (1960) famously identified the poetic function as one of the six essential functions of language, emphasizing that poetry draws heightened attention to the form of language—its sound, rhythm, and structure—rather than solely its referential meaning. In doing so, poetry functions as a natural bridge between language and music, both of which rely heavily on patterned auditory features to engage listeners. Haiduk and Fitch (2022) characterize poetry as a hybrid or intermediate system that occupies a space between the linguistic and the musical. Other examples of such systems include opera, infant-directed speech, religious chanting, and mantra—modes of expression where linguistic predictability increases while propositional content may be reduced, thus amplifying the aesthetic and emotive qualities of the stimulus. Within these systems, repetition plays a central role in fostering aesthetic engagement. Repetition at the phonological level, in particular, has been shown to facilitate the Speech-to-Song Illusion, a phenomenon where repeated speech segments begin to sound musical (Falk et al., 2014). This raises the intriguing question of whether natural languages with more regular and predictable phonological patterns—such as those with a predominance of simple consonant-vowel (CV) syllable structures—are inherently more likely to be perceived as aesthetically pleasing. Supporting this idea, Rabanus (2003) compares Italian and German and finds that Italian exhibits a CV structure in 58% of syllables, compared to just 31% in German. This phonological simplicity and rhythmic regularity may partially explain why Italian is often perceived as more melodious or musical than German.

At the segmental level, some accounts suggest that Italian is widely perceived as aesthetically pleasing because it contains very few (if any) sounds that are not shared by other European languages (Brunner, 2014). This phonological familiarity or predictability may contribute, at least in part, to the language’s perceived beauty. While this idea has yet to be empirically tested, it raises a hypothesis: could the most common speech segments—those frequently occurring across the world’s languages—possess heightened aesthetic value precisely because of their ubiquity? This notion may help explain the findings of Crystal’s (1995) study on phonological beauty in English. As mentioned earlier, Crystal examined why certain English words were perceived as beautiful based on their sound structure rather than their semantic content. He identified recurring features such as the presence of consonants like /l/, /m/, /n/, and /s/, a preference for diphthongs, initial syllable stress, and the relative absence of consonant clusters. For example, the word “melody” was rated as far more aesthetically pleasing than “gossamer.” Interestingly, many of the phonemes that Crystal identified as having the highest aesthetic index are also among the most commonly found in the world’s languages (Moran et al., 2019).

Research into the aesthetic and emotional impact of specific phonemes in poetry has produced mixed and often contradictory findings. While some studies suggest that particular phoneme classes may be associated with specific emotional tones—such as plosives occurring more frequently in poems conveying joy or positivity, and nasals appearing more commonly in sad or melancholic contexts (Albers, 2008; Auracher et al., 2010; Wiseman and van Peer, 2003)—other investigations challenge these associations. For instance, earlier work by Fónagy (1961) and later studies by Whissell (1999, 2000) propose that plosives can convey negative or harsh characteristics, while Tsur (1992) argues that nasal vowels may be linked with perceptions of beauty. More recently, Aryani et al. (2018) conducted a large-scale lexicon analysis focusing on the affective potential of speech sound and reported a number of phonetic features potentially causing the effect of sound on meaning. Specifically, they demonstrated that short vowels, voiceless consonants, and hissing sibilants provoke greater arousal and negative associations in the listener.

Despite the lack of consensus on specific sound-emotion pairings, there is an overall scholarly agreement that phonological form does play a significant role in shaping the affective experience of language used in poetry. Recent empirical studies have provided evidence that sound patterns in poetry can influence emotional responses during reading, even in silent reading conditions. Aryani et al. (2013, 2018) and Ullrich et al. (2017), for example, demonstrate that phonological properties—such as sonority, prosody, and phonotactic regularity—can evoke affective states and modulate the perceived emotional tone of a poetic text. Sonority has been an especially promising candidate as a predictor of perceived language beauty. Sonority refers to the relative loudness or acoustic prominence of speech sounds, as perceived by the human ear, and is typically organized in a hierarchy: vowels are the most sonorous, followed by glides, liquids, nasals, fricatives, and stops (Ladefoged, 2000; Parker, 2008). This gradation is crucial for understanding how language appeals to the auditory system and evokes aesthetic responses. In poetic texts, higher sonority often correlates with increased musicality and emotional resonance. For example, Studniarz (2016) illustrates how Edgar Allan Poe exploits sonorous phonemes in his poems to enhance both the rhythm and affective tone of the poem. Similarly, research in the field of neurocognitive poetics supports the idea that phonological features—especially those related to sonority—contribute to the aesthetic appreciation of language. Jacobs (2015) conducted a study on German words rated for their beauty and found that the most beautiful words (e.g., “Libelle” dragonfly) that refer to natural phenomena and concepts associated with well-being are also the words with a high concentration of sonorous phonemes. In contrast, the words with the smallest sonorous index were often related to taboo topics or bodily functions. Jacobs points out that poetry frequently uses highly sonorous words for aesthetic effects.

Kogan and Reiterer (2021) measured sonority of 16 natural languages and demonstrated that languages with high overall sonority such as Spanish and French (which usually coincides with frequent use of open syllables, fewer consonant clusters, and a preference for voiced and sonorous phonemes) were rated as particularly appealing by naive listeners who did not speak these languages. Another study by Nemestothy (2022) measured mean sonority for 29 European languages and compared it with listeners’ aesthetic ratings. The findings confirmed that more sonorous languages (e.g., the Romance languages) are judged more erotic and less orderly (more “blurry”), while ratings of beauty and status showed no robust links. In this study, cross-language ranking placed several Romance/Celtic varieties (e.g., Spanish, French, Portuguese, Breton) toward the sonorous end and many Germanic/Slavic languages (e.g., German, English, Russian, Polish) toward the less-sonorous end, though there was variability. An exploratory geographic analysis suggested higher sonority tends to cluster in southern/western Europe (consistent with climate/“acoustic adaptation” ideas), but the author cautioned that family membership and methodology limit causal claims. Overall, both works support a phonaesthetic connection: sonority relates systematically to how languages are perceived, especially along eroticism vs. orderliness scales. Seemingly vowel-heaviness (sonority direction) makes languages being perceived as more “blurry,” but nice, whereas salient presence of consonants (or consonant clusters) makes languages sound less harmonic or soft, but more structured or “orderly,” perhaps carving shapes into the sound impression. Currently, repetition at the phonological level and sonority are the most promising directions to investigate phonoaesthetics of natural languages.

## 8 Between music and language—chills, lyrics, and sound patterns

It is not uncommon for listeners to describe the sound of certain languages as “music to my ears.” Popular accounts suggest that some languages are more musical or singable with Italian often leading the list. For example, LingoDigest (2023) suggests that there are tangible reasons why Italian remains the dominant language in opera with its “clear vowel sounds and smooth consonants” contributing to easy projection in singing. Yet, the acoustic properties shared by speech and music that may contribute to aesthetic pleasure in both domains have not been empirically investigated. A notable exception is Kogan and Reiterer (2021), who examined overlaps between speech and music across acoustic and aesthetic dimensions. They found that higher tempo and compressed pitch range increase arousal in both; in speech, these cues are often judged “sexy” (with languages like Spanish, rated highly on aesthetics, tending to be faster and more pitch-compressed). Music elaborates these cues into a richer aesthetic system, whereas speech uses them more functionally and to a lesser extent. Even so, listeners often experience speech as musical, especially those with musical training. An example of a strong reaction to emotionally-loaded stimuli are chills, which are mainly researched in context of music and less in language (“phonetic chill”). Chills are fleeting bodily sensations often accompanied by goosebumps (piloerection) and described as a psychophysiological response originating in areas like the spine, neck, shoulders, or scalp. They can be triggered by emotional experiences, particularly in response to music. While music-elicited chills have been studied quite extensively (De Fleurian and Pearce, 2021), a less explored but intriguing area are the phonetic or language-elicited chills, which could potentially bridge the gap between the aesthetic pleasure of music and language.

The relationship between music and language has long intrigued researchers across disciplines, from linguistics and psychology to neuroscience and musicology. Both systems are uniquely human and share a number of structural and cognitive features, including hierarchical organization, temporal sequencing, rhythm, and the use of pitch and timbre to convey meaning or emotional nuance. Patel (2008) argues for a shared cognitive and neural infrastructure supporting music and language processing, proposing the Shared Syntactic Integration Resource Hypothesis (SSIRH), which suggests that while music and language have distinct representations, they draw on overlapping neural mechanisms for processing. Neuroimaging research studies are particularly helpful to demonstrate how shared processing resources are distributed between language and music. Functional MRI (magnetic resonance imaging) and ERP (event-related potential) studies have revealed overlapping brain activation patterns during tasks involving musical and linguistic syntax, particularly in the left inferior frontal gyrus (Koelsch et al., 2002; Koelsch, 2014; Sammler et al., 2011). Sammler (2020) points out that even though speech and music show opposite hemispheric preferences in the brain—left auditory cortex for fast temporal cues critical for speech and right for fine spectral cues critical for melody, they still rely on weighted, overlapping mechanisms across both hemispheres. Yet, the exact degree of this overlap appears to vary. Recent research suggests that it depends on factors such as task complexity, individual differences in expertise (for example, musical training), and the specific subcomponents of language or music that are being processed (Christiner and Reiterer, 2013).

One major area of overlap between language and music lies in prosody and rhythm. Studies have shown that sensitivity to musical rhythm is correlated with language-related abilities such as phonological skills and reading (Christiner and Reiterer, 2015; Gordon et al., 2015). This connection is particularly salient in developmental research, where early musical training or enhanced rhythmic perception has been associated with better language acquisition outcomes (François et al., 2013; Magne et al., 2006; Moreno et al., 2009; Moreno et al., 2011). Conversely, children with dyslexia show deficits in temporal processing, both in language and in music (Flaugnacco et al., 2015).

If speech and music are processed in similar ways, it is not surprising that certain languages might evoke emotional response in much the same way music does. Research suggests that acoustic features such as speech rate and fundamental frequency function similarly in both domains, influencing listeners’ emotional responses in comparable ways (Juslin, 2000). Speech rate, or tempo, refers to the number of units (e.g., syllables) per unit of time and serves as a temporal cue in both speech and music. Faster tempos are typically associated with high-arousal or “active” emotions like anger or happiness, whereas slower tempos correlate with low-arousal or “passive” emotions such as sadness or tenderness (Juslin and Laukka, 2003; Ma and Thompson, 2015). In fact, faster spoken languages were rated more favorably (more erotic) over slower-paced ones in an above-mentioned study comparing 16 European languages (Kogan and Reiterer, 2021). Fundamental frequency (F0), the acoustic correlate of perceived pitch, similarly mirrors emotional expression in both speech and music. Rising F0 contours often evoke active emotional states, whereas falling contours are linked to passive emotions (Cordes, 2000). Pitch variation also plays a role: in one study, greater variability in pitch heightened perceptions of joy, anger, or fear, while reduced variation was linked to sadness and subdued anger (Breitenstein et al., 2001). In Kogan and Reiterer (2021), the pattern was reversed for speech: a wide pitch range was perceived as whiny, while a compressed range conveyed energy and eroticism. Certainly, music has emotion-specific structural devices (e.g., mode, harmonic progressions) that have no direct counterpart in speech. However, some low-level acoustic parameters (tempo, F0, pitch range) are interpreted similarly in speech and in music, and this may contribute to why certain languages sound more or less appealing. These findings suggest that the “music” of language—its rhythm, pitch, and tempo—plays a crucial role in shaping emotional and aesthetic impressions. In this sense, the boundaries between speaking and singing may be less distinct than they appear.

While many studies highlight parallels between emotional expression in speech and music (Christiner and Reiterer, 2015; Flaugnacco et al., 2015; François et al., 2013; Gordon et al., 2015; Koelsch et al., 2002; Koelsch, 2014; Magne et al., 2006; Moreno et al., 2009; Moreno et al., 2011; Patel, 2008; Sammler et al., 2011), not all findings support a fully overlapping pattern (Ilie and Thompson, 2006; Krumhansl, 1990; Ozaki et al., 2024; Quinto et al., 2013). In a well-known study, Ilie and Thompson (2006) manipulated several acoustic cues across both domains. They found that certain features such as fast tempo led to similar perceptions in speech and music, but the results were different for pitch. The high-pitched speech was rated as more pleasant, whereas the same was not true for high-pitched music. This contrast suggests that listeners interpret pitch in relation to its communicative function—in speech, a higher pitch may signal friendliness or emotional warmth, while in music it can simply alter tension or brightness without conveying intent. The authors concluded that, although similar neural circuits may link acoustic cues to emotional meaning in both domains, attentional strategies likely differ. In speech, listeners prioritize verbal content, while in music, greater attention is paid to aesthetic and structural properties. Other research supports the idea of domain-specific emotional cues due to the distinct functional roles of speech and music (Krumhansl, 1990). For example, Quinto et al. (2013) analyzed pitch variability and rhythmic features in both modalities and found that while pitch variation effectively conveys emotional intent in speech, it does not function identically in music. This divergence may be partly attributed to cross-cultural variation in emotional expression. Scherer et al. (2001, 2013) describe these as “pull-effects,” where cultural norms shape how emotions are encoded and interpreted in verbal communication. Although emotional decoding across cultures tends to be relatively accurate, in-group listeners are generally better at recognizing emotions than out-group listeners (Mesquita, 2003). This was confirmed in a large-scale study by Ozaki et al. (2024) who also demonstrated culture-specific differences in how music and speech relate to each other. This study is particularly noteworthy because many of its co-authors were native or heritage speakers of the languages examined, and they created and annotated their own recordings to ensure cultural authenticity of the stimuli. That being said, globally songs are slower, higher in pitch, and use more stable pitches than speech—which was also confirmed by Sammler (2024). One can also observe a quotidian phenomenon differentiating music from speech about the urge of people to move to rhythmic music upon hearing music’s “beat,” but this rarely happens when listening to “language” in a conversation. Dancing to language is rather uncommon. Overall, these findings while acknowledging the differences between music and speech, caution against assuming a universal emotional code across domains and cultures, highlighting the complexity and context-dependency of affective communication in both speech and music.

The lack of direct acoustic analogs between speech and music complicates efforts to draw definitive comparisons between the two domains. To address this issue, Chow and Brown (2018) employed musical notation as a tool for analyzing speech melody. They converted the F0 trajectories of spoken utterances from hertz into semitones and transcribed them into relative musical scores. Their analysis revealed that, unlike music, speech tends to be atonal and exhibits only a weak form of chromaticism (the use of semitone intervals that create tension and color in music). Nevertheless, even within the limited pitch range of standard speech, language-specific melodic patterns can still be identified. Font-Rotchés and Torregrosa-Azor (2015) explored such patterns by comparing the intonation of yes-no questions in Spanish and German using the Melodic Analysis of Speech (MAS), a method grounded in the principle of phonic hierarchy and based on F0 measurements of vowel segments. While their study revealed some cross-linguistic similarities in melodic contours, it also highlighted significant differences in tonal range—so pronounced that a declarative statement in one language could be perceived as a question in the other. Similarly, Mennen et al. (2012) found notable cross-linguistic variation in F0 span, reporting that German speakers tend to use a wider pitch range than English speakers. Despite these insights into pitch range and intonation, the aesthetic value of prosodic patterns and whether they contribute to the perceived musicality of certain languages remains largely unexplored. Although some studies have addressed aspects of the aesthetic overlap between speech and music, the topic remains relatively uncharted.

## 9 Neuroscientific aspects of phonaesthetics

The perception of aesthetics across art, language, music, and other domains has received growing attention in cognitive neuroscience, particularly within the field of neuroaesthetics. This development was catalyzed by the pioneering work of Semir Zeki, whose research on the neural basis of color perception laid the foundation for studying artistic beauty in the brain (Tomohiro and Zeki, 2011; Zeki, 1999). A lively example from Zeki (1999) concerns the perception of the famous mobiles by Alexander Calder. These kinetic sculptures, which are powered either by motors or by the wind, use motion as the dominant element in the viewer’s perception. Zeki focuses on two brain areas in the visual cortex to explain Calder’s sculptures: V4, which is especially active in the perception of color, and V5, which is important for motion perception. Generally, when V4 is strongly active (for instance, when one views abstract color patterns), the activity in V5 is reduced. However, in Calder’s work—where he largely restricts his palette to black, white, and red—he unknowingly maximizes stimulation of V5 (motion). If he had used secondary colors, he would have “confused” the perceptual clarity of his mobiles. Zeki’s work initially inspired investigations primarily in the visual arts, but the momentum it generated soon broadened to encompass other aesthetic domains. As research activity expanded, scientific societies and dedicated conferences emerged, further consolidating the field. Over the past two decades, this growing body of interdisciplinary work has coalesced into what is now recognized as the field of neuroaesthetics.

Neuroaesthetics examines how the brain gives rise to experiences of beauty, pleasure, and artistic appreciation, linking neural activity to subjective aesthetic experience. Aesthetic experience is a complex, multilayered phenomenon involving perceptual, cognitive, and emotional processing. Leder and Nadal (2014) define empirical aesthetics not simply as the study of beauty or art, but as the investigation of the psychological mechanisms through which people perceive, evaluate, and emotionally respond to aesthetic objects and experiences. In their influential model of aesthetic appreciation and judgment, Leder et al. (2004) describe aesthetic experience as emerging through a sequence of processing stages: perception, classification, cognitive mastery (understanding and making sense of the stimulus), and evaluation. For example, when a listener hears an unfamiliar piece of music, they first perceive its basic sensory features—tempo, pitch, and timbre. Next, they classify it as belonging to a certain style or genre (e.g., classical, jazz). As the piece unfolds, they attempt to achieve cognitive mastery by detecting patterns, anticipating musical structure, or linking it to prior knowledge. Finally, they evaluate the experience, forming a judgment about whether the piece is expressive, engaging, or beautiful. This sequential interplay of perception, understanding, and interpretation is central to Leder et al.’s account of how aesthetic experiences unfold. Later work (Leder, 2013) emphasizes that contextual, stylistic, and individual factors shape aesthetic judgments, highlighting that such experiences are not universal but influenced by expertise and culture. Two listeners hearing the same unfamiliar piece of music may form entirely different aesthetic judgments depending on their cultural background, musical training, and familiarity with the style. Thus, the sense of beauty arises from a dynamic interplay between bottom-up sensory input and top-down cognitive appraisal These intertwined mechanisms are supported by distributed neural systems integrating sensory data with higher-order cognition. As Chatterjee and Vartanian (2014) note, aesthetic experience is not a passive reaction but an active, interpretive process combining perception and thought.

Building on this understanding, neuroimaging studies have identified brain regions that support aesthetic evaluation. In particular, areas of the so-called default mode network (DMN),—a network typically active during wakeful rest, mind-wandering, and self-generated thought—plays a key role. Regions within this default mode network, most notably the medial prefrontal cortex and posterior cingulate cortices, are consistently engaged during reflective and emotionally meaningful aesthetic judgments. Because the DMN is active when individuals are not focused on external tasks but instead engage in introspective, self-referential thought, its involvement highlights the internal, personal nature of aesthetic experience. The activation of these regions during aesthetic appreciation suggests that such experiences rely not only on processing external sensory features but also on memory, emotion, and self-related cognition (Vessel et al., 2012). This evidence supports the view that aesthetic appreciation is deeply introspective and shaped by internal states rather than purely by external stimulus features.

While the neural mechanisms of aesthetic experience have been widely explored in visual art and music, much less is known about aesthetics in speech. Specifically, research has only begun to address how the sounds of language themselves, independent of meaning, can evoke affective or aesthetic responses. A few studies in German have examined this so-called sublexical (referring to constituent parts of a word) affective potential. Ullrich et al. (2016) demonstrated that certain phoneme clusters associated with high-arousal or negative meanings elicit early neural responses during lexical tasks, suggesting that some sounds carry inherent emotional weight. Such early neural responses are brainwaves that reflect rapid, automatic emotional evaluation of stimuli—in this case, the speech sounds. In a complementary fMRI study, Aryani et al. (2018) presented listeners with spoken German nouns that were grouped into high vs. low sublexical arousal (how exciting or agitated they sound), while carefully matching their lexical arousal (emotional meaning). Words whose sound profile was rated as more arousing—typically containing more abrupt onsets, sharper consonant clusters, or greater acoustic energy—produced stronger activation in the brain, even though participants were only asked to listen attentively and were not explicitly judging emotion. At the textual level, Menninghaus et al. (2017) analysed a corpus of German poems and showed that sublexical “basic affective tone”—quantitative measures derived from the distribution of phonemes and sound patterns, explains additional variance in readers’ ratings of a poem’s overall affect (e.g., friendliness, sadness, spitefulness) and aesthetic qualities, over and above lexical meaning alone. For instance, poems that were perceived as more spiteful or harsh tended to contain a higher proportion of phoneme sequences with a negative or high-arousal sound profile, whereas “friendly” poems exhibited more sonorous and euphonious patterns. Together with Ullrich et al. (2016), these findings support the idea of phonological iconicity: systematic correspondences between the sound structure of words (or texts) and their affective impact, such that listeners respond emotionally to the sound pattern itself, not only to meaning.

Research on poetic language provides another clear bridge between linguistic and musical aesthetics. Wassiliwizky et al. (2017) showed that emotionally powerful poetry can elicit aesthetic chills—the same full-body goosebumps often reported with music. Their fMRI results revealed activation in classic reward-related brain regions, including the nucleus accumbens and orbitofrontal cortex, which are central components of the brain’s pleasure and motivation circuitry. However, the precise spatial activation patterns differed from those observed in music-elicited chills (Blood and Zatorre, 2001; Salimpoor et al., 2011): poetic chills tended to involve additional areas linked to meaning-making, mental imagery, and autobiographical memory. To make this more intuitive: while both poetry and music can feel deeply moving, poetry draws its emotional force not only from sound but from understanding, i.e., the evocation of images, memories, and personal associations. The brain signatures mirror this; poetry engages reward regions plus areas that support comprehension and reflection, whereas music-induced chills rely more strongly on the dynamics of sound, tension, and anticipation. This distinction highlights a fundamental difference in aesthetic processing: poetic language inherently carries semantics, whereas music does not. This sets poetry apart from the experience of listening to unfamiliar languages, where the listener cannot access meaning and the response is driven almost entirely by sound patterns: rhythm, intonation, phonetic texture. Early evidence suggests that such unfamiliar speech may activate neural pathways that blend music-like auditory processing with responses to novelty, ambiguity, and pattern detection, making it a promising direction for future research.

These findings spanning iconicity, emotional sound symbolism, and the reward dynamics of poetic language suggest that aesthetic responses to speech emerge from interactions between sound structure, emotional processing, and meaning. Yet they also raise a deeper question: what general computational principles guide the brain’s aesthetic responses across different domains such as music, language, and even unfamiliar speech?

Recent theoretical advances in neuroaesthetics, particularly predictive coding frameworks (Friston, 2010; Pearce et al., 2016), offer a unifying lens for integrating these observations. Predictive coding posits that the brain is constantly attempting to anticipate incoming sensory information; aesthetic pleasure arises when stimuli strike the optimal balance between fulfilling expectations and gently violating them. In other words, the most engaging experiences are neither fully predictable nor fully chaotic, they sit in the sweet spot where the brain’s predictions are challenged just enough to maintain curiosity without overwhelming the system.

This framework helps make sense of aesthetic responses to both music and linguistic sound. For speech, it explains why unfamiliar languages can feel intriguing: the listener recognizes some patterns (prosody, rhythm, phonotactics) but encounters enough novelty to keep prediction error low yet engaging. Kogan and Reiterer (2021) provide empirical support for this idea: listeners rated unfamiliar languages as more aesthetically pleasing when they contained a blend of familiarity and novelty, capturing what the authors termed an “exotic touch.” This aligns with findings from musical aesthetics, where listeners tend to prefer musical systems and compositions that balance recognizability with surprise (Eisentraut, 2012). Together, these strands of evidence suggest that predictive coding may serve as a cross-domain principle guiding aesthetic appreciation in both language and music, shaping how listeners respond to familiar versus novel sound patterns.

The neuroscience of phonaesthetics proper is still in its very infancy. Findings so far suggest that aesthetic pleasure (whether evoked by language, poetry, or music) relies on shared neurocognitive principles, including the default mode network, predictive processing, emotional resonance, and the interplay of novelty and familiarity. At the same time, each domain introduces distinct constraints: semantics and cultural context in language, and formal structure and tonality in music. In short, our brains seem to use similar reward and prediction systems to find beauty in words, sounds, and melodies alike. Understanding both the overlaps and the divergences between these domains will be essential for developing an integrated account of aesthetic experience across human communication systems.

## 10 Practical implications and future directions

Phonaesthetics research has implications that reach far beyond the academic study of sound symbolism or linguistic beauty. Understanding how sound evokes aesthetic and affective responses offers practical applications in education, language learning, marketing, artificial language design, and neuroaesthetics. For instance, evidence that phonaesthetically pleasant sounds enhance memorability and processing fluency (Matzinger and Kosic, 2025a) could inform second language instruction and vocabulary learning by integrating sound-based aesthetic awareness into pedagogy. Knowing that familiarity with languages—be it through first, second or foreign language knowledge—is such a strong appeal and reputation booster, could influence educational, political and mass media policy makers for future language use and curriculum choices. Similarly, in branding and advertising, phonaesthetic principles are already intuitively used, but empirical findings now allow for more systematic and cross-linguistic design of brand names, product labels, and slogans that align sound, meaning, and emotional impact. The aesthetic affordances of language sound also hold promise for therapeutic and artistic practices—from poetry and voice training to speech therapy and emotional expression—where sound-based affect could be deliberately harnessed to support wellbeing and communication.

Future research should continue to bridge linguistic, psychological, and neuroscientific approaches, deepening our understanding of how phonaesthetic judgments emerge at cognitive and neural levels. Neuroimaging and psychophysiological methods could clarify how aesthetic pleasure from linguistic sound relates to mechanisms of predictive coding, reward processing, and emotional resonance, and whether these mechanisms differ from those engaged by music. Expanding beyond WEIRD contexts and exploring cross-linguistic and cross-cultural variation will be crucial for developing a global model of linguistic aesthetics. Additionally, as new technologies enable the creation of artificial voices and languages—from AI-generated speech to constructed languages (conlangs)—phonaesthetics offers both an ethical and aesthetic framework for ensuring that such systems respect human affective sensitivity to sound.

Ultimately, the future of phonaesthetics lies in integration and application: combining empirical rigor with creative insight to better understand why certain sounds move us, how they shape our perception of language and identity, and how this knowledge can be responsibly applied in an increasingly sound-designed world.

## 11 Conclusion

Like the mythological creature “phoenix” resurrected and rising from the ashes after destruction, phonaesthetics, once a very old, almost mythological philosophical topic itself (Krapinger, 2014), later on dead, then a marginal topic in linguistics (Köhler, 1929; Crystal, 1995), is now re-emerging and reborn as a rich interdisciplinary field linking language, cognition, perception, emotion, and aesthetics. This review has traced how the study of sound beauty—long dismissed as subjective—has gained new empirical grounding through advances in cognitive science, neuroaesthetics, psycholinguistics and linguistic typology. Despite a growing interest, it is still not that widely distributed and echoed as much longer established fields. While related, phonaesthetics focuses on the aesthetic properties and apperception of language sound, while sound symbolism studies the motivation for meaning behind sound, thus making phonaesthetics a distinct old-new research field, still being much smaller in publication impact and output magnitude (Figures 1, 2) than its bigger brother: sound symbolism (Figure 3). Contemporary research demonstrates that sound is not merely a transparent vehicle for meaning but an aesthetic medium in its own right, shaping how language is learned, remembered, and valued.

From David Crystal’s foundational work to modern studies on sound symbolism, memory and phonaesthetic word learning, brand naming, phonaesthetic non-word studies, constructed languages research, and cross-linguistic comparisons of sound patterns, neuro-cognitive studies on lexical arousal, the evidence consistently points to an interplay between inherent acoustic features and culturally mediated norms. Familiarity (mostly in the form of foreign/second language knowledge) has been singled out as one of the most powerful sources and forces of phonaesthetic appraisal (Anikin et al., 2023; Reiterer et al., 2020); to be familiar with certain languages gives them a sound-aesthetic advantage. However, this individual, but also largely culturally mediated factor, is not an all-explaining variable. Furthermore, it would be equally interesting to find out the exact reasons that lead to this foreign language expertise and thus “feeling of familiarity,” being multifacetedly engrained in political, historical, societal/cultural, but also individual etiologies.

While speech-sound aesthetic preferences also vary individually—across speakers, voices, contexts, cultures, personality types, and musicality profiles (“individual differences”)—research increasingly points to cross-culturally recurring patterns in certain phonological/phonetic domains. Features such as sonority, phrasal rhythmicity (isochrony patterns), vowel distribution, prosody, speech melody, articulation speed, and specific phonemes or sound combinations (e.g., approximants versus voiceless fricatives; Lev-Ari and McKay, 2022) suggest that our sensitivity to sound beauty may rest on shared cognitive and emotional mechanisms. For example, overall findings propose that voiced consonants (as in /b/, /m/, /n/), approximants (like /l/, /w/), smooth prosody, fast speech, high vowel share, high sonority increase pleasantness, while back vowels, voiceless fricatives, slow and irregular speech (melody) reduces it (Nemestothy et al., 2024). A weak global trend was even found for tonal languages to be less favorably rated (Anikin et al., 2023). Across European languages, higher sonority correlates with higher erotic and lower “orderliness” ratings, forming a north/east-to-south/west aesthetic gradient (Nemestothy, 2022; Reiterer et al., 2020; Kogan and Reiterer, 2021). Research on Tolkien’s Elvish, Klingon, and Dothraki or non-words experimentally designed to sound “harsh” (as in the context of “swearing”) confirms that intentional sound design successfully evokes emotional impressions (pleasant vs. harsh; Mooshammer et al., 2023). Words judged as aesthetically pleasing are remembered better; intriguingly, moderately beautiful words are rated most appealing (Matzinger and Kosic, 2025a). Repetition, rhythm, and sonority are also exploited in poetry and poetic use of language to express feelings. Speech can emotionally affect listeners like music and preliminary evidence shows that spoken language can evoke chills and physiological arousal, pointing to a shared aesthetic-emotional mechanism.

Controlled speaker studies show that both phonetic cues and social prestige stereotypes affect how listeners judge languages’ attractiveness. Both individual attitudes and collective and discursive language ideologies, continue to modulate these responses and effects at the same time, reminding us that aesthetic judgment is both embodied and social. An overall impression of this research lets us cautiously conclude that these aesthetic experiences attached to speech sounds, patterns or even languages, are more grounded in the social and individual sphere than in language-internal properties (inherent value, see also Figure 4). Yet, we do not know the ratios and the last word has not been spoken. The avenue of future research in this area is still long.

Reviving phonaesthetics therefore means more than cataloguing pleasant sounds; it invites a renewed inquiry into why sound matters—in how we form linguistic identities, evaluate languages, and experience speech as art. By bridging linguistics with psychology, neuroscience, and the humanities, phonaesthetics offers a framework for understanding language not only as a system of communication but also as an aesthetic and affective phenomenon.

In this light, language itself should join the ranks of the traditional arts as a legitimate subject of aesthetic evaluation alongside music, painting, and literature. Its sounds, rhythms, and prosodic patterns can elicit beauty, emotion, and meaning just as powerfully as melody or color. Recognizing this expands the boundaries of both aesthetics and linguistics, positioning language as an art form that speaks not only to the mind but also to the senses and emotions. Thus, the “phoenix” of phonaesthetics rises again, not merely as a study of linguistic beauty, but as a call to reimagine language as one of the human arts.
